# A case of GABAR antibodies in schizophrenia

**DOI:** 10.1186/s12888-016-1157-2

**Published:** 2017-01-10

**Authors:** Ida S. Haussleiter, Klaus-Peter Wandinger, Georg Juckel

**Affiliations:** 1 Department of Psychiatry, LWL University Hospital, Ruhr-University Bochum, Alexandrinen Str. 1, 44791 Bochum, Germany; 2 Institute of Laboratory Medicine, University of Luebeck, Luebeck, Germany

**Keywords:** GABA, Autoimmune, Encephalitis, Schizophrenia

## Abstract

**Background:**

In the last couple of years, schizophrenia was often discussed as autoimmune disease. Several antibodies were suspected, but so far there has been no proof of Gamma-aminobutyric acid (GABA) receptor antibodies in patients with schizophrenia.

**Case presentation:**

In this case report we present a 21-year old woman with schizophrenic symptoms, who showed anti-GABAB1 antibodies when screened by a vast recombinant neurology mosaic on Human Embryonic Kidney Cells 293 (HEK293) cells. The young woman presented with various psychotic symptoms as well as speech and motor ataxia, with the neurological signs starting in childhood.

**Conclusion:**

A hypofunction of the GABAergic system is a possible cause of severe schizophrenic symptoms. Postmortem studies proved this hypothesis by showing dysfunctional GABAergic interneurons in various brain areas. Therefore one should always think of an immune-mediated pathogenesis as well memory impairment and behavioral changes co-occur with frequent seizures.

## Background

Schizophrenia is a severe polygenic and heterogeneous psychiatric disease with onset in early adulthood. According to the Diagnostic and Statistical Manual of Mental Disorders schizophrenia is characterized by profound disruption in cognition and emotion, psychotic manifestations and negative symptoms. The causes of schizophrenia have been the subject of much debate and the underlying pathomechanism remains so far unknown [1]. Intrinsic inflammatory and immunologic processes without sufficient repair mechanisms seem to be involved [2].

Glutamate is a key player in the mediation of schizophrenic symptoms and by non-competitively antagonizing the glutamate N-methyl-D-aspartate (NMDA) receptor with substances such as ketamine, phencyclidine, or Dizocilpine (MK-801) in animals [3], neurological and psychiatrics symptoms similar to those in humans can be produced. They comprise positive (hallucinations, delusions, thought disorder) and negative (anhedonia, blunted affect, avolition) symptoms of schizophrenia, as well as movement dysfunction, autonomic instability and seizures [4]. The described psychomimetic effects of NMDA antagonists have been attributed to pre-synaptic functional receptor blocking in thalamus and frontal cortex, causing a decreased Gamma-aminobutyric acid (GABA) release [5]. GABA has a major inhibitory function in the central nervous system and modulates neuronal activity and working memory via inhibitory prefrontal neurons. There are three GABA receptor types (A, B, C), of which GABA_B_ is a G-protein coupled transmembrane receptor consisting of two subunits (B1, B2). Subunit B1 is responsible for the transmitter binding as well as for the general receptor function, whereas B2 ensures the transmembrane localization and the G-protein activation. A direct antagonistic binding to the GABA receptors (GABAR) and dysfunctional inhibitory cascade could imitate the same schizophrenic symptoms as a decreased level of the transmitter GABA itself.

The role of GABAR has already been shown in several immunohistochemical experiments in which reduced immunolabeling of GABAR subunits occurred in different brain regions (hippocampus, prefrontal cortex, inferior temporal cortex, and entorhinal cortex, lateral cerebellum) of schizophrenic patients [6, 7].

In case of a GABA_B_ receptor antibody mediated encephalitis patients usually present with a classical limbic encephalopathy with seizures. Magnetic Resonance Imaging (MRI) findings typically show an increased signal in the medial temporal lobes, and Cerebrospinal Fluid (CSF) shows an elevated lymphocyte cell count. The animal model of GABA_B_ receptor antagonism resulted in similar symptoms such as seizures, memory deficits, anxiety, and affective dysregulation in rodents [8]. Lancaster et al. assessed and serologically screened 15 patients with suspected paraneoplastic or immune-mediated encephalitis as well as 104 controls for neuronal antibodies with a transfected HEK293 assay. Anti-GABAB1 antibodies were found in all patients and half of them had tumors, mainly small cell lung cancer [9]. However anti-GABA_B_ receptor encephalitis also develops without cancer association and resembles other central synaptic autoimmunities. The presence of CSF antibodies in patients with both neurological and psychiatric deficits has been examined [10, 11]. Whereas anti-GABA_B_ receptor antibodies were frequently found in previously seronegative patients with encephalitis and small cell lung cancer, they did not occur in patients with isolated cerebellar ataxia [10].

## Case presentation

The 21 year old woman was admitted to our psychiatric ward as an emergency for the first time in 2010, reporting hallucinatory voices as well as persistent visual hallucinations (command to jump out of the window, blood running down the walls) and tried to harm herself by repeatedly smashing her head against the wall. Moreover, she demonstrated psychomotor agitation and suffered from alcohol intoxication with 1.98 promille. The psychotic symptoms remained after detoxication and the patient reported to have suffered from negative symptoms such as apathy and depressed mood during the previous six months. The patient was diagnosed with paranoid schizophrenia according to the international classification of disease (ICD-10), which is commonly used in clinical routine as well as psychiatric research. During the following three months the patient was admitted two more times, always in a floridly psychotic state with self-inflicted injuries and intoxicated with alcohol. Antipsychotic treatment comprised ziprasidone and quetiapine with no satisfactory effect. The patient was treated with ziprasidone only for a couple of weeks and in a low dose. Continuous was with quetiapine for almost two years in an initial dose of 500 mg and a maintenance dose of 100 mg daily, both without considerable effect. Dosage was reduced due to the patient’s pregnancy.

The patients’ first psychiatric contact occurred 2004 at the age of 14, when she was brought to a child psychiatrist because of unstable interpersonal relationships, self-consciousness, identity, and behavior difficulties. Auto-aggressive tendencies started at the age of 12. Because of acute distress, difficult adjustment to social situations and somatic manifestations, three more hospital admissions to child and adolescent psychiatry became necessary subsequently, all lasting but a few days.

According to her mother the patient was delayed in speech and motor skills and attended a school for learning-disabled children. At the time of consultation, a psychomotor retardation was still evident. The patient complained of frequent headaches. The findings of the neurological exam indicated an ataxia in speech (dysarthria) as well as motor coordination. In pediatric physical assessment somatosensory evoked potentials (SEP) recording (N. tibialis) and electromyography (EMG) (M. tibialis anterior) were normal, electroencephalography (EEG) was normal according to age. Brain MRI and angiography did not show any space-occupying lesion or vascular malformation. There were no detectable alterations of the purine and pyrimidine metabolism and electrophoresis of lipoproteins did not reveal any pathological results either.

One sample of serum was collected and serum was isolated by centrifugation (2500 x g/ 10 min) and kept at −80 °C until completion of recruiting phase. Serum was subsequently sent for diagnostic workup to the authors’ lab (Luebeck, Germany), and tested for the presence of antineuronal antibodies by means of a commercial immunofluorescence assay (Autoimmune Encephalitis Mosaic, Euroimmun, Luebeck, Germany).

**Fig. 1:**
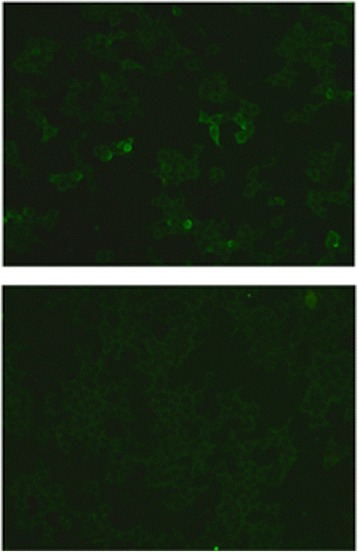
HEK cells co-transfected (*above*) and non-transfected (*below*) with cDNA for GABA receptor (B1). Serum of patient bound to the surface of cells transfected with GABARs, Antibody titre 1:320, Irani Score 2

Slides were incubated with patient samples at a starting dilution of 1:10. After incubation for 30 min at room temperature, the slides were rinsed with a flush of Phosphate Buffered Saline (PBS)-Tween and incubated in PBS-Tween for at least 5 min. Bound antibodies were labelled using Fluorescein-conjugated goat anti-human immunglobulines IgG, IgA or IgM antibodies for 30 min and washed as described before. Samples were classified as positive or negative based on the intensity of surface immunofluorescence of transfected cells in direct comparison with non-transfected cells and control samples. Central nervous system (CNS) tissues were assessed for the presence of characteristic staining patterns indicative of the presence of antibodies against neuropil or other neuronal antigens in the patient’s sample. In a dilution of 1:320 the patient’s serum still showed antibody binding to GABA_B_ surface antigen in the assay (Fig. 1).

The patient refused plasmapheresis to remove the antibodies. Further diagnostic including CSF samples (to confirm the presence of receptor antibodies) and chest x-ray (to rule out a Small Cell Lung Cancer (SCLC)) were not performed for ethical reasons, since the patient accidentally got pregnant.

The Ruhr-University Bochum ethics committee approved the analysis (3250–08), and written informed consent was obtained from the patient.

## Conclusions

Several auto-antibodies against neurotransmitters and their receptors (such as antiserotonin, antinicotinic and antimuscarinic receptor antibodies) have been found in serum or CSF of schizophrenic patients, thus suggesting an autoimmune etiological connection [12]. Anti-dopaminergic receptor antibodies or inhibition of the transmitter itself do not seem relevant [13], but the GABAergic control of this dopaminergic system seems an important factor in the course of schizophrenia [14]. A hypofunction of the GABAergic system is a possible cause of severe schizophrenic symptoms. Postmortem studies proved this hypothesis by showing dysfunctional GABAergic interneurons in various brain areas [15]. Therefore one should always think of an immune-mediated pathogenesis as well memory impairment and behavioral changes co-occur with frequent seizures. The prevalence of anti-GABAB1 antibodies in clinically defined tested cohorts (limbic encephalopathy) was 0.14 and 0–0.04 in healthy and illness cohorts respectively [9, 16].

So far there has been no proof of GABA receptor antibodies in patients with schizophrenic symptoms. In this case report we presented a young woman with schizophrenic symptoms, who was screened by a vast recombinant neurology mosaic on HEK293 cells and showed anti-GABAB1 antibodies. The young woman presented with various psychotic symptoms as well as speech and motor ataxia.

The clinical coexistence of neurological and psychiatric features in patients with encephalitis has been known for quite some time, but recently cell surface antibodies have been found in patients with so far purely psychiatric symptoms. For example, specific receptor antibodies (anti- Voltage-gated potassium channel complex (VGKC), anti-N-methyl-D-aspartate receptor (NMDAR), anti-a-amino-3-hydroxy-5-methyl-4-isoxazolepropionic acid receptor (AMPAR)) were found in (young) patients with first episodes of psychotic symptoms [17, 18]. In some cases of clinical evident schizophrenia autoantibodies to cell surface antigens such as the GABAB receptor might emerge. In these cases, further diagnostics to rule out a tumor or another infection have to been undertaken, since symptoms may regress under immunotherapy or tumor removal.

The current case is only a single description of an association in a patient that was not neurologically treated. There can therefore be no conclusion about the general relevance of these antibodies in schizophrenia, but the need for screening further patients is evident.

## Abbreviations

AMPAR: α-amino-3-hydroxy-5-methyl-4-isoxazolepropionic acid receptor
CNS: Central nervous system
CSF: Cerebrospinal Fluid
EEG: Electroencephalography
EMG: Electromyography
GABA: Gamma-aminobutyric acid
GABAR: GABA receptors
HEK293: Human Embryonic Kidney Cells 293
ICD: International Classification of Disease
IgA: Immunoglobulin A
IgG: Immunoglobulin G
IgM: Immunoglobulin M
MK-801: Dizocilpine
MRI: Magnetic Resonance Imaging
NMDA: N-methyl-D-aspartate
NMDAR: N-methyl-D-aspartate receptor
PBS: Phosphate Buffered Saline
SCLC: Small cell lung cancer
SEP: Somatosensory evoked potentials
VGKC: Voltage-gated potassium channel complex
